# Containing Hunger, Contesting Injustice? Exploring the Transnational Growth of Foodbanking- and Counter-responses- Before and During the COVID-19 Pandemic

**DOI:** 10.1007/s41055-022-00099-y

**Published:** 2022-03-19

**Authors:** Charlotte Spring, Kayleigh Garthwaite, Andy Fisher

**Affiliations:** 1 Laurier Centre for Sustainable Food Systems, Wilfrid Laurier University, Waterloo, Canada; 2 Department of Social Policy, Sociology and Criminology, University of Birmingham, Birmingham, UK; 3 Portland, Oregon USA

**Keywords:** Foodbanks, COVID-19, Right to food, Philanthropy, Household food insecurity

## Abstract

COVID-19 caused levels of household food insecurity to spike, but the precarity of so many people in wealthy countries is an outgrowth of decades of eroding public provisions and labour protections that once protected people from hunger, setting the stage for the virus’ unevenly-distributed harms. The prominence of corporate-sponsored foodbanking as a containment response to pandemic-aggravated food insecurity follows decades of replacing rights with charity. We review structural drivers of charity’s growth to prominence as a hunger solution in North America, and of its spread to countries including the UK. By highlighting pre-pandemic pressures shaping foodbanking, including charities’ efforts to retool themselves as health providers, we ask whether anti-hunger efforts during the pandemic serve to *contain* ongoing socioeconomic crises and the unjust living conditions they cause, or *contest* them through transformative pathways to a just food system. We suggest that pandemic-driven philanthropic and state funding flows have bolstered foodbanking and the food system logics that support it. By contextualising the complex and variegated politics of foodbanking in broader movements, from community food security to food sovereignty, we reframe simplistic narratives of charity and highlight the need for justice-oriented structural changes in wealth redistribution and food system organisation if we are to prevent the kinds of emergency-within-emergency that we witnessed as COVID-19 revealed the proximity of many to hunger.

## Introduction

Watching Donald Trump and Boris Johnson fail to preside over the unfolding tragedy of COVID-19, as millions lost their jobs and spectacular lines formed outside foodbanks, felt despairingly, dully predictable. Leaders’ ostrich approach^1^ (“it’ll go away! Just wash your hands!”) echoed the indifference and neglect of recent decades of rolling back protective entitlements. Four decades of neoliberalism have withered states’ capacities to uphold their duties to people, and people’s individual capacities to protect themselves have in turn been hampered by years of austerity. Many hearts have been hardened by the concretised ‘common sense’ that healthcare, housing and other basic provisions should be treated as market, not public, goods. As researchers of poverty, food, and health in Britain and America, we’ve witnessed worsening prospects for precarious and working people for many years, in the wake of 2007-8’s Global Financial Crisis and, across the ‘special relationship’, neoliberal populist leaders elected who’ve captured sections of the working class by appeals to chauvinistic scapegoating (Fraser 2019; Saad-Filho 2020). The COVID-19 crisis looks set to exacerbate poverty levels and further widen pre-existing health inequalities (Bambra et al. 2020; Gundersen et al. 2020).

Those who cannot afford to meet their basic needs in increasingly marketised economies have been especially vulnerable to overcrowding, enforced mobility and destitution during COVID-19, rendering some people at far greater risk to the virus than others. Leaders’ heel-dragging responses portended a predictable response to the ever-larger numbers of people facing extreme hardships in the spring of 2020: an upswell in charity food provision in the US and UK, our respective home countries. This highlights a growing transnational trend of masking the failures of welfare systems through their voluntary sector appendages, including the use of food charity. While many scholars have noted upswells in foodbanking on the heels of recessions and crises since the 1970s (Riches 2018), the extent of private and public funding flows to food charities in the wake of the COVID-19 pandemic suggests, we will argue, a turbocharging in the global rollout of this approach to containing the contradictions of corporate excess alongside the cumulative effects of neoliberal austerity. This has also reinvigorated contestation around root causes of, and structural solutions to, inequality and hunger. While acknowledging the difficult and important work of food charity providers, we ask whether pandemic responses to hunger serve to *contain* ongoing socioeconomic crises and the unjust living conditions they cause, or *contest* them through transformative pathways to a just food system.

Despite significant critique of food charity as anti-hunger response (Lohnes and Wilson 2018; Poppendieck 1998; Tarasuk 2001; van der Horst et al. 2014), we have seen its persistent expansion across wealthy nations. Riches (2018, p.163) argues that “the 35-year story of the global spread of US-style charitable foodbanking in the rich world’s embattled welfare states is of promises to feed all those ‘left behind’ with surplus food, while its corporate partners benefit from neoliberal economic policies fuelling a prolonged crisis of income poverty and widening inequality”. Riches notes that predatory multinational agrifood interests have captured governments responsible for protecting the Human Right to Food, producing what Fisher (2017) has characterised as a ‘hunger industrial complex’. (Neo)colonial, corporatised food regimes transcend national boundaries, while global governance of food has become a key target of monied interests and Big Philanthropy (Fakhri et al. 2021; McMichael 2009). These historical precursors have, we will suggest, led to foodbanking being positioned as a key response during the pandemic, in increasingly internationally-coordinated ways that we feel demand an internationalist response.

We will show how US-style foodbanking’s (pre-pandemic) international expansion and normalisation was driven by four key trends: welfare retrenchment; the outsourcing of states’ responsibilities to uphold the Right to Food; food overproduction ‘balanced’ with aid; and the interests of ‘Big Food’ in charitable redistribution. These have solidified international foodbanking as a mode of ‘containment’ of system contradictions and injustices (Heynen 2010). Critics have long argued that foodbanking upholds an ecologically damaging and economically unjust food system. Nevertheless, foodbanking’s growth trajectory has incorporated some of these critiques, for example by shifting towards models of healthier food provision and other service upgrades. Some charities have shifted further, towards political advocacy for wealth redistribution and the Human Right to Food, sometimes in alliance with broader social movements seek to transform excessive food production systems that utilise charity to absorb surpluses.

The pandemic has reinvigorated such debates and shifts. As states have re-asserted their capacities to respond to income crises through public assistance, the demands placed upon food charities and mutual aid providers have re-surfaced questions around the appropriateness of food-based versus income-based responses to the endemic food insecurity that preceded COVID-19. In this time of reckoning with systems of economic exploitation based on colonialism, patriarchy, misogyny, anti-Black racism, capitalist accumulation and ableism, demands have resurfaced that basic needs to be met not by well-meaning volunteers, but by universal income and service guarantees.

This review paper is co-written by members of the Global Solidarity Alliance for Food, Health and Social Justice (#RightsNotCharity), thinking and acting to critique the international expansion of US-style foodbanking (Cohen 2020). It draws on our experiences of researching and campaigning in North America and the UK over several decades, observing and challenging how the UK and other counties have adopted practices from North America, where food charity has long been institutionalised. We argue that COVID-19 has further exposed the limitations of charitable models (and the relative importance of government entitlements). We suggest genuine alternatives that could ensure access to food as a basic human right (rather than primarily as a source of profit or charitable gift), highlighting advocacy for ‘cash-first’ approaches in the UK that combine localised governance with universal entitlements.

In contrast to previous analyses of food charity’s relationship to economic crises, COVID-19 presents a unique opportunity to question links between food systems, infectious disease, and dietary inequality in ways that force us to examine the sustainability and equity dimensions of dominant responses; in this case, corporate foodbanking as a mode of managing food system contradictions. Globalised, industrial food systems have contributed to pandemic risks, including COVID-19 (Smith 2020). COVID-19’s impacts can be viewed as a ‘syndemic’, implying the way two or more ‘diseases’ (broadly understood) interact in context, producing potentially worse respective outcomes (Horton 2020, unpaginated). COVID-19’s interactions with food insecurity and diet-related illness provide an example of this. As a social determinant of health, food insecurity is linked to non-communicable disease but also, relatedly, to different groups’ and individuals’ susceptibility to infectious disease. COVID-19 mortality is associated with poor diet and diet-related illness, which track along lines of (often racialised) class inequalities that were intensified for many during the pandemic (Connors et al. 2020; Golestaneh et al. 2020). Working in poorly-paid jobs, such as in grocery stores, meat processing plants, or elder care homes, increases one’s exposure to infection and, where employment is precarious and unstable, to further risks of food insecurity (Clapp and Moseley 2020). In the US, pay rises for “essential workers,” often the low-wage workers with high exposure to COVID, were temporary at best (or simply denied). The pandemic should thus encourage us to consider systemic causes of, and solutions to, these interconnected vulnerabilities and their relation to food system ethics and histories; preventing the uneven effects of disease outbreaks requires tackling underlying socio-economic disparities (Horton 2020).

The paper proceeds as follows: we first provide a snapshot of food insecurity during the pandemic, and the response. We analyse the rise of the dominant ‘containment’ response of internationally-expanding US-style foodbanking^2^, via four key drivers. We then examine how food charity has been shaped by internal and external contestation in response to critiques, broadly differentiating between efforts to improve, or to eradicate, foodbanking. External pressures include the influence of movements for ecologically-informed food justice. We argue that these pre-pandemic trends conditioned the variegated food responses seen during the pandemic, from philanthropically-driven charity expansion to mutual aid and campaigns for income-maximisation. Distinguishing between containment or contestation of hunger, we conclude by asking whether these food-related responses to the pandemic look set to disrupt or to reaffirm prior trends towards corporate-backed and globally-expansive containment.

## COVID-19 and Food Insecurity

Here we provide an initial snapshot of pandemic impacts on household food security and the food charity response.

COVID-19 and ensuing lockdowns unleashed rising demand for food assistance in the US and UK. UK advocates reported sharp rises in food aid demands, including a 110% rise in food parcel delivery reported by the Independent Food Aid Network (IFAN) from February to December 2020 (IFAN 2020). Foodbanks in the US were overwhelmed with demand, prompting many to have to switch procurement tactics at a time of instability in supply chains (Lakhani et al. 2020). US estimates of food insecurity rates more than tripled in the first months of the pandemic (Wolfson and Leung 2020). Household food insecurity levels and food access issues have changed over the course of the pandemic in complex and uneven ways in different places and for different groups, but many have consistently struggled to afford and access good food, particularly communities of colour and those with disabilities (Food Foundation 2021; Loopstra 2020; O’Hara and Toussaint 2020).

Many larger foodbanks became awash with private donations from concerned onlookers and corporate donors in the first months of the pandemic. As unemployment numbers soared, Jeff Bezos added $25bn to his personal wealth in the first couple of months of lock-downs (Kelly 2020). Bezos’ wife became the richest woman on the planet by virtue of divorcing him, and donated millions to foodbanks (Ali 2021), as celebrity campaigns urged the public to do the same.

The alarming rises in demand for charity in the wake of COVID-19 must be seen in the context of conditions that preceded the pandemic, particularly the extent of food insecurity and inadequacy of public welfare prior to the crisis (HLPE 2020). As O’Hara & Toussaint (2020, p.5) note, “﻿Food insecurity…emerges as both a cause and symptom of COVID-19”.

Many food charity affiliates know that- even improved- charity cannot solve hunger (Patrick et al. 2020). For more progressively-inclined charitable food providers, responding to COVID-19 required abandoning hunger-preventative programming and advocacy, and returning to a 1980s-era prerogative of meeting the immediate urgency of hunger using available resources. As crisis-within-crisis, the precarity that COVID-19 has unleashed emerges not out of thin air, but from decades of defunded welfare infrastructures and neoliberal culturing of devolved service provision to private charity (Warshawsky 2010).

## Trends Driving International Expansion of Us-Style Foodbanking

The precipitous rise in foodbank use and the charitable food aid sector in the UK is, not unlike its evolution in North America, a result of ever-weakening welfare provision hampering people’s efforts to provide for themselves and their families (e.g. Lambie-Mumford 2013; Garthwaite 2016). The replacement of entitlements by charity represents an ‘old normal’ of hegemonic neoliberalism, eroding the ‘common’ sense that all should share in the spoils of economic growth (Fraser 2019). The past four decades’ austerity policies, described by Gilmore (2007) as “organised abandonment”, have frayed formerly robust social welfare nets, resulting in millions living in poverty in the UK and North America.

While many critiques of UK foodbanking have drawn on North American literature, less attention has been paid to the comparability of different contexts (exceptions include Hebinck et al. 2018, Lambie-Mumford 2013), and the potential for allied critiques and movements. In this section, we analyse the internationalisation of foodbanking, particularly its US origins and its spread to the UK. Charitable food aid has become normalised in both regions, but in somewhat distinct ways that demand contextualisation of its origins and discourses in both regions. We do not wish to simple restate existing critiques of food charity, many of which are specific to the US (DeLind 1994; Henderson 2004; Warshawsky 2010; Fisher 2017). We also recognise that the UK has a long and distinct history of managing inequality through charity, including charitable food. Here, we describe four interrelated trends driving foodbanking as key containment response to food insecurity, particularly in wealthy countries: welfare retrenchment; the outsourcing of states’ responsibilities to uphold the Right to Food; food overproduction ‘balanced’ with aid; and corporate interests in charitable redistribution.

There has been a lack of explicit attention to the causes, nature and comparability of US-style foodbanking across the globe^3^. Our analysis draws on our experience of the US and UK where foodbanking has taken root where declining welfarism has exacerbated wage and welfare precarity (Riches 2018), but foodbanking is expanding in many parts of the world, especially where corporatized agribusiness has expanded. Particular geographical and political contexts shape the extent and nature of food insecurity and charitable food (Warshawsky 2010), and important analytical lessons will come from attending to specific articulations of governments, charities and food industries in different places over time^4^. However, we observe explicit attempts to expand foodbanking internationally, including by umbrella networks such as the Chicago-based Global Foodbanking Network (GFN) and their corporate sponsors. The UK provides an example of how the US model is being scaled out under the tutelage of such institutions (Garthwaite 2016).

### Driver 1: Neoliberal Welfare Retrenchment

Against a backdrop of state rollback, the US’ Personal Responsibility and Work Opportunity Reconciliation Act (PRWORA) of 1996 made substantial cuts to social welfare programs (Allen 1999; Poppendieck 1998). ﻿Campbell et al. (2015, p.2) note that this resulted in a major change for ‘emergency’ food assistance from serving primarily jobless individuals towards people in chronic need of food assistance: “many client families (more than 54 percent in 2014) contained a working member”. In the UK, post-recession austerity policies since 2010 have driven ever-growing levels of household food insecurity and charitable provision (Caplan 2017; Fabian Society 2015).

Welfare has been retooled as a means of disciplining labour forces (Dickinson 2016; Heynen 2010). Intensifying conditionalities of social security have been identified as a key cause of rising food charity use in the UK (Loopstra and Reeves 2015). Allen (1999, p.118) points out that the US federal SNAP programme (formerly ‘food stamps’) was “originally developed largely through the self-interested rent-seeking character of economic agents rather than social welfare”. An ‘able-bodied adult without dependents’ is entitled to only three months’ access to SNAP in a single three-year period if not working in formal employment or a workfare program. Part of the PRWORA welfare reforms, this provision removed the “implicit guarantee that no one need starve…there is no longer a publicly-funded unconditional right to food” (Poppendieck 1998, p.284). As state support dwindled, private charities and their supply of redistributed food grew.

### Driver 2: Charitable Outsourcing

Forty years of welfare retrenchment have been accompanied by an outsourcing of provisioning from governments to other sectors. Such ‘hands-off’ policymaking-through-grantmaking responsibilises communities rather than enacting state governments’ duty to uphold the Right to Food (Guthman 2008). This has been accompanied by a ‘restructuring’ of popular narratives around responsibility and deserve: Daponte and Bade (2006, p.677) note Reaganite views of foodbanking as the “optimal solution to hunger”, premised on the ‘win-win’ of appeasing anti-hunger advocates while servicing the food industry. Over 45 million Americans access food charity via the Feeding America network annually, so that even as enrolment for SNAP benefits has risen sharply (Weinfield et al. 2014), apolitical approaches remain central to imaginaries of ‘tackling hunger’.

The visceral, affective hold of learning of neighbours’ food precarity, and of wanting to respond with gifts of food and voluntarism afforded by food charity (which Poppendieck 1998, described as a “moral safety valve”) helped to nourish the rapid growth of American foodbanking. Feeding America is the third-largest charity in the US (Lohnes n.d.), its scale underpinned by low-paid or voluntary labour and the fundraising capacity of community-level organisations (Tarasuk and Eakin 2005). Similarly, the rapid growth of the UK’s emergency food aid sector since the turn of the millennium has been accompanied by public discourses through which charitable food provision has become socially acceptable as a way to manage what in the UK has commonly been described as ‘food poverty’ (Garthwaite 2016; Fabian Society 2015).

### Driver 3: Managing Food and Labour Surpluses with Aid

Food aid has long been tied to the surpluses generated by monopolistic agribusiness, enabling ‘soft power’ and labour disciplining (McMichael 2009). As a net exporter of food, America has long used foodbanking to uphold farm commodity prices (Poppendieck 1998). Here, we note the specificity of American foodbanking’s embeddedness in US food value chains that should be borne in mind when comparing with other countries. Foodbanking is historically entwined with US agrifood policy that incentivises industry participation in the charitable food regime. Poppendieck (1998) locates the origin of US food charity in New Deal-era compromises, where it served as discursive and infrastructural means to relieve politically embarrassing contradictions of agricultural surpluses alongside hunger. Following revelations of government food stockpiles- as he was signing welfare cuts into being, Reagan created The Emergency Food Assistance Programme (TEFAP) to redistribute agricultural surpluses through charitable networks. The influx of food to ad-hoc charitable efforts in the early 1980s resulted in “a dramatic increase in responsibility for the food pantries…[and] encouraged existing charities to add food distribution to the list of services they already provided to the poor” (Daponte and Bade 2006, p.677).

While the ‘T’ in TEFAP originally stood for ‘Temporary’, Poppendieck (1998) notes how this was changed as these ad-hoc efforts formalised into more permanent infrastructures that persist to this day. State and federal programs pay for roughly one-quarter to one-third of food handled by foodbanks. Despite the ‘responsibility’ imposed upon America’s charities, TEFAP continues to provide “very little support for logistics, infrastructure or additional staffing capacity” needed to process and distribute food surpluses; leaving them to manage “economic crises within their own locales” (Lohnes and Wilson 2018, p.5). Crucially, Lohnes (2020) argues that, counter to the idea that expanding food charity is simply a function of greater demand from needy people, “﻿supply-side dynamics are key to understanding why the demand for emergency food is maintained through food charity”. While food assistance programmes like SNAP have been regularly threatened with cuts, TEFAP and its relationship to foodbanking remains a “critical appendage” of industrial food chains’ reliance on manufactured scarcity to maintain profitability (Lohnes 2020).

The UK does not produce the agricultural excesses that have enabled food supplies to be managed through US charity networks via TEFAP. UK government support for charitable surplus redistribution has been limited, although we later explore changes to this light of COVID-19. Nevertheless, concerns over farm-level waste have spurred calls for surplus produce to be channelled charitably as well as commercially (Forsey 2014). Public problematisations of UK food wastage highlight how the UK’s import-dominated food system also commands significant caloric surpluses, premised on the undervalued labour of food workers (Feedback Global 2015; Nally 2011). While lacking US foodbanks’ integration with agriculture, UK local authorities have at times diverted resources to the stockpiling of food to shore up food charities’ capacity. For example, food security concerns in the wake of the Brexit referendum at times saw food charities align their own concerns for sustainability with the interests of ‘national security’ (Quinn 2019).

The global rollout of foodbanking mirrors efforts to privatise, industrialise and consolidate food systems (Warshawsky, forthcoming). The charitable re-channelling of food surpluses and the replacement of cash entitlements with food transfers has been documented across OECD and countries experiencing the ‘supermarketisation’ and consolidation-corporatisation of food value chains (Nally 2011; Watson and Battersby 2019). Many such regions have seen changes in state food policy from delivering the Human Right to Food via entitlements, towards governing trade and regulation, a process increasingly fuelled by food’s financialisation (Dowler and O’Connor 2011; Riches et al. 2014).

### Driver 4: (State-subsidised) ‘Big Food’ Interests in Food Charity

The assumed responsibility of the charitable sector for communities’ food and other basic needs invites discussion of whose interests are best served by charitable food (Caraher and Furey 2017). Winne (2008, p.175) notes that America’s “anti-hunger policies have always been joined at the hip with attempts to help farmers, promote national security or serve another interest or constituency”. Foodbanking benefits corporate food donors reputationally, and by managing wastage, while volunteers and charitable infrastructures struggle to cope with managing the demands of donors and food-seekers. Lohnes and Wilson (2018) demonstrate how burgeoning food flows that financially benefit corporate food donors and their wasteful practices induce precarity on charitable distribution systems that are supposedly ‘sharing’ redistribution costs.

The international growth of charitable food redistribution has also been stimulated by legislation to incentivise charitable donation of surplus foods, reflecting the diversion of state functions and resources to subsidise private externalities under neoliberalism. Such policy is reflected in American foodbanking practice e.g. Feeding America endorses ‘Good Samaritan’ laws to protect firms from liability. Lindenbaum (2016) notes this as just one of many state-backed ‘gifts’ accruing to food corporations, including tax breaks, trade policy, state research and subsidies; paving the way for crisis bailouts to which we return later. France and Italy have introduced legislation to impel and incentivise charitable donation (Arcuri 2019; Mourad and Finn 2019), with such efforts underway in the US (New York State 2019).

The UK’s lack of such a law does not appear to constitute a barrier to surplus donation (Downing et al. 2014). Nevertheless, throughout 2015 Global Foodbanking Network member and foodbanking enterprise FareShare lobbied for legislation to financially incentivise surplus donation, which it argued would better implement food waste management according to the ‘food recovery hierarchy’^5^ (Anderson 2015). Their campaign lobbied for public money to offset redistribution costs for businesses that they argued incentivised excess food to be sent for animal feed or anaerobic digestion rather than diverted to charity. A UK Parliamentary Bill in 2016 proposed “incentives for individuals, public sector bodies and private sector companies to implement and encourage observance of the food waste reduction hierarchy” (House of Commons 2016). Little mention was made in these campaigns about the potential costs borne by charities for the eventual distribution of wasted food (Lohnes 2020). Some foodbanks impose costs that sometimes hard-stretched community organisations must bear in order to receive food (Forsey 2014). The UK government announced a series of grants enabling redistribution organisations to cover logistical costs, amounting to over £15m and enabling rapidly-expanding foodbanking organisations to offer financial rewards to major food businesses^6^.

This section has highlighted several important factors when comparing factors favouring foodbanking in different countries. To summarise the containment approach, the growth of food charity is underwritten by a neoliberal food regime founded on private profit, land enclosures for monopolistic commodity production and the enactment of geopolitical ‘soft power’ (Holt-Giménez and Shattuck 2011). Food’s commodification, agri-food consolidation, and the responsibilisation of individuals and communities for their own subsistence, have coalesced in the global spread of charitable foodbanking as a ‘permanent emergency’ response within ‘food bank nations’ (Riches 2018). It should be noted that many of these factors bear longer histories than the label ‘neoliberal’ implies, but rather have deep roots in charitable institutionalisation, ideas of ‘deservingness’ and in the long durée of capitalist food system development, beyond foodbanking simply being a US import. In recent decades, charity has been rescaled and normalised as a response to persistent economic crisis, following retrenched welfare entitlements and declines in labour power. Many food providers, including those in our Alliance, are well aware of critiques, including foodbanking’s alignment with capitalist accumulation (DeLind 1994), its chilling effect on hunger-preventative policy (Henderson 2004), its exacerbation of stigma (van der Horst et al. 2014), its limited food offer (Poppendieck 1998), and withdrawal of state governments from responsibility for ensuring household food security (Warshawsky 2010).

These drivers of hunger containment through foodbanking have conditioned the ways in which food charities have been positioned as a key response to COVID-19. Before analysing this response, we suggest how some foodbanks have attempted to respond to critiques, prefiguring different possible futures- and contestations that charities could embrace, in light of COVID-19’s revelation of both the extent of need and limitations of charity.

## Towards ‘better’ Foodbanking, or no Foodbanking? Pre-Covid Contestation from within and without

### Contestation From within Foodbanking: Improving Provisioning vs. Exit Strategies

This section discusses evidence that some foodbanks have responded to critiques through reforming their service and, less commonly, by seeking more radical solutions to ending hunger, thus ‘doing foodbanking out of a job’. McIntyre et al. (2016) note that the inherent tension between ‘improving’ and ‘eliminating’ foodbanking may present challenges for allied critiques that would lend policy weight to the foodbanking exit strategies advocated by Poppendieck (1998). This section relies heavily on the US, as this is where the majority of challenges and changes have been documented, sometimes in place-specific ways, but we make note of where the UK also shows evidence of pre-pandemic shifts.

Given COVID-19’s highlighting of the health implications of ecological and socio-political conditions, it is worth considering foodbanks’ previous attempts to assert themselves as solutions to health inequalities. Health has risen as a discourse of self-justification for foodbanking. Amidst decades-long concerns around the links between diet-related illness and poverty, and accusations of providing unhealthy food (e.g. Poppendieck 1998), some US foodbanks have attempted to improve their impacts through the lens of health, which Campbell et al. (2015) describes as an evolution towards ‘nutrition-focused’ foodbanking (for a European example see Hebinck et al. 2018). Yet focusing on nutritional content alone fails to address the industrial model of food production that produces pandemic risks (Smith 2020), nor the socio-economic inequities determining food access.

This nutrition-focused shift responded not only to criticisms of inadequate food offerings, but also to declining availability of excess manufactured products, leading some foodbanks towards purchasing, rather than relying upon donations. Foodbanks located in areas of high agricultural productivity may be able to negotiate contracts with local farmers. Others collaborate with local healthcare providers, giving out ‘healthy’ food boxes to clients to test against diabetes outcomes (Campbell et al. 2015). Others invest in community gardening or other food production activities, although these do not seem to be denting overall flows of surplus commodities (Vitiello et al. 2015).

Such shifts reflect the purchasing power of well-established charities receiving significant private sponsorship (Fisher 2018), and sometimes also incentives by private healthcare providers seeking future healthcare cost-savings. In other words, profit motivates activity that might nourish the bodies of low-income people. Lohnes (n.d.) questions how health problems like diabetes are placed in competition with hunger as problems to be solved by foodbanks, risking obscuring broader drivers of health inequalities. Additionally, focusing on food quality alone ignores sociopolitical determinants of health. The time and care expended by certain foodbanks should be treated as expressions of improvement within a system that many staff realise is imperfect, with small improvements potentially leading to more systemic changes (Rosenthal and Newman 2019).

Similar discourses around health and food assistance have grown in prominence in the UK. Prior to the pandemic, statistics bearing out rising demands and need for charitable food had been increasingly accompanied by efforts to interrogate the future of foodbanking. Some food charities attempted to increase availability of fresh fruit and vegetables, or provide welcoming atmospheres that attempt to reduce the potential stigma of accessing food assistance (Saxena and Tornaghi 2018). Others were experimenting with ‘shopper’ models to nuance the uneven giver/receiver dynamic inherent to food charity (Fisher 2017; Spring et al. 2019; van der Horst et al. 2014). Some US providers were attempting to harness food for community building and economic development e.g. Food Shift in Oakland and DC Central Kitchen capture surplus produce for use in developing catering business, training and employing formerly incarcerated clients. Community Food Centres Canada’s model links ‘healthy’ food distribution with skills-building, empowerment, political advocacy, and community building (Levkoe and Wakefield 2011).

Despite these improvements, surplus food redistribution has remained entrenched – conceptually and materially- as an acceptable solution to hunger and waste (Arcuri 2019). If we accept that long-term solutions to pandemic risk must address socio-ecological factors that worsened COVID-19 outcomes in the wealthiest countries, such systemic issues cannot be resolved through simply improving food charity. Many charities realise this and, prior to the pandemic, were seeking more radical solutions. Lohnes and Wilson (2018, p.16) suggest that “as feeding lines continue to grow, many donating money and labor to this network are beginning to question their commitment in light of injustices they perceive in the wider food system”, prompting a search by some for more structural solutions that also address ecologically-harmful food production (Dickinson and Lohnes 2019).

Four decades of service have rendered some volunteers tired, frustrated, and advocating for more radical changes to foodbanking. Lohnes and Wilson (2018) cite ageing foodbank volunteers as ‘waste workers’ questioning their long-term labouring to provide food charity while perceiving broader systemic injustices. ‘Freedom 90’, for example, was a self-appointed ‘union’ of foodbanking volunteers in Ontario, Canada. It grew from community organising around neoliberal welfare retrenchment in the 1990s (Taylor 2016). Leveraging the political capital of pensioners carved Freedom 90 a unique, irony-tinged mission: to do themselves out of unpaid charity work by age 90 (the eldest member notably passed the age of 90) (Spring 2016). Freedom 90’s activism aimed utilise this subject position to mobilise fellow volunteers and wider publics and re-frame hunger and charity in terms of social (in)justice and state responsibility, in line with Riches' (2018, p.172) call for “public support and political will for the right to food and social justice agenda informed by collective solidarity”. Critique of charity volunteerism has also been increasingly focussed on its gendered and racialised/ing dynamics (de Souza 2019; Guthrie 2020a).

UK commentators have long observed US foodbanking and urged caution around importing its more regressive aspects (Hawkes and Webster 2000). Far from accepting food charity’s explosive growth, UK practitioners and researchers have debated its ethical and political implications. Writing of the political potentials of UK food charity spaces, Williams et al. (2016) point to their contestatory discursive atmospheres, where perceptions of charity and need can be confirmed or upturned, highlighting potentials for critical, cross-Atlantic alliances. Critical understandings of UK food charity as an import of US-style foodbanking have gained ground (Garthwaite et al. 2019).

The UK foodbanking community had thus begun searching for ‘exit strategies’ prior to the pandemic, with a nascent consensus coalescing around calls for an end for foodbanks’ existence. A former CEO of the UK’s largest charitable food franchise, the Trussell Trust, said “every town should have [a food bank]” (Lambie-Mumford 2013). However, current CEO Emma Revie has expressed a desire to put Trussell “out of business”, insisting that foodbanks should not be a long-term solution (Butler 2018). To this end, the Trust has been publicly advocating around for improvements to the UK’s main welfare reform, Universal Credit, a means-tested benefit for working-age people working-age on a low income. They showed that almost half (49%) of foodbank referrals were linked to delays in Universal Credit payments (The Trussell Trust 2019). Uses of data for advocacy by charities themselves hints at shifts towards recognition by foodbankers that food-based solutions can only ever be partial^7^. These calls were amplified as dominant COVID-19 responses threatened to further entrench sticking-plaster solutions (Goodwin 2020).

Food charities’ pathway towards exit strategies must include volunteers on the front lines but, critically, those who are experiencing food insecurity, to develop a shared analysis of root causes of ‘rich world’ hunger, with an aim of creating “humane welfare encounters” that can “begin to challenge both the form of, and need for, food aid itself” (May et al. 2019, p.1271). Changing containment narratives, frames and stores must include those most affected by poverty in developing any solution or response. UK efforts include the programming priorities of the UK’s ‘Food Power’ programme and the Independent Food Aid Network. Alameda County Community Food Bank (ACCFB) provides an example of advocacy by charity workers; its advocacy coordinator attended government budget hearings and described vocally refusing to be positioned as an excuse for welfare cuts^8^, as well as convening a board of foodbank clients (Spring 2016). We were inspired by the foregrounding of expertise-by-experience in campaigns such as Canada’s Put Food in the Budget, whose coordinators recognised that individuals’ analyses of their own contextualised stories develop over time, and may need close support before participating in public campaigns (Spring 2016).

### Contestation from Outside of Foodbanking: Community Food Security and Food Justice

The US in particular bears a strong history of radical food activism targeted at inadequate state provisions and the charitable containment of poverty. With progressive foodbank leaders in our network increasingly looking to these movements for messaging and praxis that could build genuine alternatives to charity, contestation involves forming alliances with movement coalitions seeking transformative and systemic change to harmful food systems (Patel and Goodman 2020). Here, we review Community Food Security, food justice and food sovereignty as overlapping contestatory discourses that can contextualise the impacts and future orientations of pandemic-prompted food mobilisations.

### Community Food Security (CFS)

A key US-founded response to contest the shortcomings of foodbanking, and root causes of hunger, has been Community Food Security. Hamm & Bellows (2003, p.40) define CFS as “a situation in which all community residents obtain a safe, culturally acceptable, nutritionally adequate diet through a sustainable food system that maximizes community self-reliance and social justice”. This reconceptualization of hunger, away from an individual pathology to be palliated with charity, “embodies a community-based and prevention-oriented framework that focuses on both immediate and long-term food security” (Allen 1999, p.119). It attempts to critique the corporate food system and assert forms of democratic control over ecologically-viable food systems. CFS thus incorporates a more radical diagnosis of food system problems and social justice-oriented prognosis of potential solutions (Hamm and Bellows 2003). Contrastingly, issues of how, and where, food is produced, have not been broadly challenged by foodbanking institutions such as Feeding America (understandable, given aforementioned points about the imbrication of corporate and charitable imperatives).

The re-scaling of food security to the community level in CFS attempted to posit opportunities for greater food democracy; for participation of people in localised food systems through efforts such as urban agriculture and nutrition education. CFS envisages ﻿people meeting their food needs without the need for emergency charity and emphasising self-reliance. However, it has been critiqued for its potential to reify and romanticise ‘community’, recalling broader critiques of localism in ‘alternative food’ discourse; Allen (1999, p.120) notes the contingent, ideological and constructed character of ‘community’ that “provides opportunities for some and constrains those for others”. Historically, as Slocum (2006, p.330) argued, “community food organizations do not connect the dots among white privilege, institutionalized racism, their community food work and the larger food system”. Our own anecdotal evidence suggests that this situation has been changing for the positive in the past five years, while we recognise the enormity of structural barriers that remain at play (O’Hara and Toussaint 2020).

UK-based critiques of community-based development cite governments’ devolution of political problem-solving: “﻿it smacks of the self-help ethos, involves vanishingly small resources and can be encouraged without at the same time having to admit to the existence of poverty” (Leather 1996, p45; see also Spring and Biddulph 2020 on tensions between mutual aid and charity). However, CFS has left a legacy of radically-minded community food organisations focusing on poverty reduction alongside fairer and ecologically just food systems that can put pressure on mainstream foodbanking operations and foster connections across sectors, issues and places (Moragues-Faus and Sonnino 2019). Many of these include a strong emphasis on anti-racism that has been bolstered during COVID-19.

Finally, Patel (2011) notes the risks of cooptation of grassroots food assistance efforts. Allen (1999, p.126) notes that “﻿ironically, it is the industrialized food system that has reduced class differences in food consumption”, but its cooptation of organic food, for example, re-imposes nutritional inequalities based on differential ability to pay in marketised systems (Guthman 2003).

### Radical Food Justice and Food Sovereignty

Also recognising the shortcomings of charity, a food justice lens highlights structural barriers to both food access and food sovereignty, rooted in class and race struggles in US cities (Alkon and Agyeman 2011). The global food sovereignty movement originated in peasant farmer struggles but has become a global ‘movement of movements’ (Holt-Giménez and Shattuck 2011). We are mindful of the specific socio-historical contexts and struggles that shaped these concepts when making comparisons with the UK (Clendenning et al. 2016), but we celebrate the rich traditions of radical food activism founded in courageous opposition to multiple forms of socio-economic inequality and discrimination, but that seek not only to abolish harmful systems, but build up better ones (Gilmore 2007; Heynen and Ybarra 2021).

Food justice advocates call for fairer distribution of the benefits and harms of food system dynamics through transforming the uneven power relations that characterise the ways in which food, and the means to produce and acquire it, are managed. The commodification of food, and the histories that underwrite this, are widely understood to produce first world hunger; food justice thus requires naming and transforming the “capitalist, patriarchal and racist logics that produce hunger and food insecurity” (Heynen et al. 2012). While speaking truth to structural oppression, food justice attends to discursive and spatial dimensions of food systems through practices of food sovereignty and community food security (ibid.). Recent uprisings against white supremacy and systemic racism have bolstered anti-hunger responses that challenge racist histories and policies that generate uneven food outcomes for poor people of colour (Chennault et al. 2019; Jones 2019).

Food justice provides both lens and tools for scholarship and activism that recognises historical unevenness of food access along class, race, gender and other lines of difference (Alkon and Agyeman 2011). Many food providers cannot simply expect national governments to repair welfare provisions, given decades of state neglect. Historically, movements including the Black Panther Party distributed food as mutual aid, given racialised and otherwise marginalised groups’ systematic immiseration (Heynen 2009; Patel 2011). Rather than assume the capacity (and willingness) of existing governments to answer advocates’ tireless calls for social protection, these food-based movements are predicated on a prefigurative politics, often initiated by communities of colour, to rebuild food systems from the ground up, enacting a refusal of reform narratives. A food justice and/or food sovereignty lens can help to frame groups’ demands for self-determination over foodways while highlighting the structural conditions that shape these (Alkon et al. 2020).

Dixon (2015) proposed food justice as a lens through which food charity volunteers be “epistemically positioned” to advocate through ‘counter-stories’. These challenge individualising narratives of personal responsibility, foregrounding instead structural causes of food insecurity such as racism, food worker wages, and welfare policy, and of corporate food excess such as subsidies, agribusiness lobbying and so on. While the genesis of food justice is specific to the racialised struggles of food movements in the US, such epistemic re-framings can thus apply to other neoliberal contexts where the stigmatisation of welfare demands contestation (Baker et al. 2020).

Such re-framings offer ways to shift anti-hunger praxis towards countering economic and environmental injustice. *Closing the Hunger Gap* (CTHG), a growing network of food providers, offer a series of potential narrative shifts to effect organisational transformation towards social justice-oriented models^9^. We are aware of the practical challenges involved in shifting the direction of large and magnanimously-funded foodbanking institutions, and the risks that progressive narratives be co-opted or used as rhetorical cover for broadly unchanged practices. CTHG has developed shared analyses around the need for structural transformation through organisation focus on ﻿race, privilege, and inequity, which can educate newer ‘food bank nations’ like the UK (Powers 2016). More research is needed to examine how far narrative shifts and stated commitments (e.g. to ‘do ourselves out of business’) translate to programmatic and public policy-oriented changes, and to monitor whether justice-oriented gestures are not dwarfed by continued expansion of business-as-usual (see Swords 2019; Vitiello et al. 2015 for laudable examples). We also note the need to attend to food sovereignty and Right to Food movements that have shifted policy and affected material livelihoods in countries from Brazil to India (Hossain et al. 2014; Rocha 2016), solidarity with whom will be essential in countering the globalising ambitions of corporate foodbanking (Warshawsky, forthcoming).

While many efforts have been made to characterise the political complexity and multiple motivations of actors within both radical and charitable anti-hunger responses, we recall Allen’s (1999) suggestion that traditional food programs- including federal supports such as SNAP- and community food security projects can “work together to overcome the forces that have produced food insecurity” (Allen 1999, p.127). Responses are needed at local, national and international levels, she argues. Vitally, ﻿she concludes that, “if public assistance benefits were sufficient or incomes were sufficiently high, there would be no need for food pantries” (p.126).

Foodbanks’ more progressive programmatic and policy changes can achieve certain justice-oriented outcomes, but do not necessarily overcome class, race and other inequities built into charitable food provision. Beyond ‘telling different stories’, the exit strategy narrative risks entrenching the palliative, reformist idea that hunger can be eliminated by making a few twinges without systemic change. We recognise the risk for co-optation of radical language and narratives that allow food charities to appear motivated by transformational goals while maintaining a status quo. However, we recognise the potential of (re)framing to widen the scope of what is seen as possible at different levels of intervention, from interactions within charity spaces to halls of power.

## COVID-19 Responses: Containing or Contesting Business-as-usual?

Having described trends shaping foodbanking praxis in the UK and US, we consider how these have shaped responses to food insecurity since the pandemic began, and whether they can be seen as containing or contesting hunger, taking our cue from Heynen (2010)’s comparison of foodbanking and food-based mutual aid.

### Containment

State and private funding for foodbanking has been turbo-charged in response to COVID-19. Towards summer 2020, the UK government’s food and agriculture department (Defra) announced funds of £16m and $63m to support food distribution charities^10^. Such commitments raise questions over how this further institutionalisation can, if at all, be scaled back in a post-COVID-19 context (Goodwin 2020).

US state funding for foodbanking preceded the pandemic, as we showed earlier. In 2018/9, the Trump administration committed to purchasing $2.6 billion-worth of commodities for foodbank distribution to help mitigate the impact of foreign tariffs on American farmers (USDA 2020). These foods come on top of the usual $300-$500m-worth of commodities and cash through TEFAP, $200m-worth of tax credits for foods donated by corporations to foodbanks, and tax relief and other programs administered by many states.

These flows have intensified following COVID-19. In May 2020, USDA approved $1.2 billion in contracts for food providers to distribute boxes of produce, meat and dairy to emergency food providers (UDSA 2020). Some anti-hunger advocates initially perceived this effort as a Trojan Horse to pilot the much-maligned “harvest boxes” proposed by the Trump administration, in which SNAP recipients would only be able to use one half of their benefits at grocery stores, while the other half would come in the form of boxes of pre-chosen commodities (Fisher 2018)^11^, posing distribution challenges for foodbanks. These funds are but a portion of the administration’s COVID-related commitments to the foodbanking industry, which include $3 billion in box distributions, plus $1.6 billion in food and cash (USDA, 2020b). The Biden Administration has ended the box scheme, in favour of embedding produce purchases (with an optional preference for locally grown) into the TEFAP program (Walljasper 2021). In contrast, public assistance through unemployment insurance and pandemic payments have lent weight to Allen’s (1999) claims of the centrality of public assistance, over food charity, in preventing food insecurity during crises (Raifman, Bor, & Venkataramani, 2021). Further reforms to welfare eligibility and adequate wages are also required to overcome the limitations of an ‘overburdened’ charitable food system and temporary pandemic measures (Wolfson and Leung 2020; Zack et al. 2021).

The federal response has been matched, if not in scope but intent, by numerous US states which provided cash or facilitated purchases of surplus food by foodbanks. For example, the Oregon legislature granted $8 million to Oregon Food Bank to meet demand (KGW 2020), while California expanded an existing private sector initiative to transport surplus produce from packinghouses to foodbanks (State of California 2020). These new programs grew from existing foodbank support programs^12^ (Fisher 2017). These responses reflect the ‘healthier foodbanking’ transition but arguably have allowed wealth extraction by the biggest farmers and distributors and thus may exacerbate systemic injustices in food production and access (Guthman 2008).

Private charitable donations skyrocketed. As-yet unpublished research by one author reviewed financial statements of the top 25 US foodbanks and found an overall rise of 349% in cash income from 2019-2020, translating to an average 59% gain in net assets, belying any pre-pandemic claims to be ‘putting ourselves out of business’. Net assets of the top 4 anti-hunger groups in the US^13^ rose by 169% over the same period; given the demonstrable impact of government income-relief on actually preventing hunger, these influxes of cash beg questions about their origins: corporate donors likely lack genuine understanding or interests in systemically addressing hunger, but during a crisis that actually benefitted many private businesses, feel compelled to donate to non-profit organisations, reinforcing neoliberal trends of replacing public goods with private charity. Witnessing this alarming rush of philanthropy towards food charity, our Alliance responded with an open letter to new philanthropic entrants to food charity, exhorting donors to invest in approaches committed to long-term food system change and rights-based approaches to food insecurity that can prevent- rather than simply manage- the symptoms of inequality (Fisher 2020; Patrick et al. 2020; RightsNotCharity 2020).

COVID-19 reduced the capacity of many foodbanks engaged in nutrition-focused, ‘client-choice’ models and other attempts to improve charitable provision. COVID-related pressures on food supplies and lockdown restriction have made it harder for charities to be discerning in their provision and have forced established food providers to scale down more ambitious programming in order to meet basic demands for food. Connors et al. (2020, p.4) note that even those aware of available food charity in the UK avoided using this due to anticipated stigma and a sense that these should be left for “people who need them most”. Rather than a sense of shared vulnerability (‘in this together’), charity remains a vector of othering, while poverty induces shame that can prevent people from seeking assistance or engaging politically to demand their rights to basic needs security. Food provider members of our Alliance described a sense of ‘returning to the 1980s’, when North American foodbanking was expanding in an ad hoc fashion (Poppendieck 1998).

These pressures have also prompted reflection about wider food system sustainability and the need for radical transformations (Goodwin 2020; The Food Foundation 2020), just as food support systems are being overwhelmed (Loopstra 2020; Power et al., 2020). As noted, some providers are asking serious questions about the sustainability and ethics of charity models, and forming alliances to advocate for approaches that address both food insecurity and ecologically-damaging agri-business models, to which we now turn.

### Contestation

Here we ask whether COVID-19 may have also strengthened rationales and motivations for radical shifts away from food charity that contains, but does not prevent, precarity.

We have seen renewed problematisation of food charity in media discourse since the pandemic (e.g. Lakhani 2021). This sits in an ambivalent relationship with widespread UK support for food charity; for example, footballer Marcus Rashford has both donated significantly to foodbanking organisations and campaigned alongside those organisations to demand greater government support, particularly for children at risk of hunger. Many food providers are questioning anew the way charitable food distribution has been normatively positioned as the most appropriate response to COVID-19-induced food insecurity^14^. While some providers have focused on food distribution alone during the pandemic, others’ responses reflect the influence of movements for food justice and sustainability by engaging in advocacy, community development or redefining their metrics of success. Some food providers remain ambivalent, exploring and watching before jumping into untested waters. As the Global Foodbanking Network seeks to expand foodbanking through influencing other countries to adopt food policies that favour surplus food redistribution, we see an acute need to turn ambivalence into action (Garthwaite, Fisher & Spring, 2019).

The Human Right to Food, allied with social movements articulated around food justice and food sovereignty, offers a lens to encompass economic and other forms of oppression (Fakhri et al. 2021; Human Rights Watch 2019). Our ‘#RightsNotCharity’ Alliance grew rapidly from the onset of the pandemic, with some of us privileged to shelter in place and develop virtual transnational connections as frontline members reported the escalating crises they were facing. Our Alliance comprises practitioners, advocates, and researchers challenging rising reliance on private philanthropy and transnational corporate foodbanking to manage hunger and poverty. It highlights structural vulnerabilities to poverty, hunger, and foodbank usage, including (dis)ability, age, ethnicity, and gender. In North America, in particular, this means dismantling embedded racism across sectors (de Souza 2019), and shining a light on these practices in a UK context (Power 2019). Food provider members share experiences of shifting from further infrastructural growth towards centring poverty-reduction and dignity in foodbanking culture. Since the onset of the pandemic, one Arizona-based member foodbank successfully instigated a fair wage campaign^15^ while putting in place steps to reduce its reliance on surplus food interception, bucking the trend of many sector leaders’ reluctance to contest, for example, the preferences of corporate boards and donors (Henderson 2004).

The post-COVID rise in demand upon UK food providers led growing calls from IFAN, alongside academics (Patrick et al. 2020), to consider prioritising a ‘cash-first’ approach to food insecurity i.e. helping people access the financial support they need without needing to turn to emergency food provision, through local advice and support (Whitham 2020). In response, the Scottish Government made a commitment to a ‘cash-first’ approach via one-off crisis grants, part of ongoing consultation to "End the Need for Food Banks” (Scottish Government 2021). ‘Cash-first’ approaches also involve local community engagement, such as referral pathway leaflets and recommendations for reducing barriers to emergency crisis grants (Marshall 2020), with the aim of preventing the further institutionalisation of emergency food aid and offering dignity and choice.

The reliance of food charity on volunteers has presented challenges during COVID-19, as older and vulnerable volunteers have needed to self-isolate (Power et al., 2020). McKendrick and Campbell (2020) report that emergency food provision has expanded in Scotland in the wake of COVID-19, and demand is expected to rise alongside concerns for sufficient labour and funds to maintain provision. Recalling Freedom 90’s efforts to leverage their essential role as (unpaid) charity volunteers, the increased burden on volunteers led some to collaborate on an open letter to the Scottish government, demanding cash-first solutions to COVID-19-induced food precarity^16^.

Interim cash supports, both in the US and UK, must not be allowed to distract from the need to repair universal safety nets (Marshall 2020). But they offer a localised alternative to charity alone. Brown & Tarasuk (2019, p.1) note how Canada’s Child Benefit (CCB), introduced in 2016, has been shown to have “disproportionately benefited families most susceptible to food insecurity”, emphasising the importance of income transfers to help people meet their basic needs. Therefore, considering an approach based on the provision of cash and local assistance, rather than food aid as the immediate emergency response, could offer possibilities for those receiving the lowest incomes. Similar calls for income-based solutions have been raised in North America (Leddy et al. 2020; McLinden et al. 2020; Wolfson and Leung 2020).

Networks of neighbourhood-level ‘mutual aid’ prompted discussions of ‘normalised precarity’- seemingly-secure jobs and infrastructures have been lost or damaged, with an unevenly shared sense of vulnerability in light of government inaction (Mendenhall 2020). While some saw such responses as an indictment of state neglect, others saw them as welcome reminders of neighbourliness, though some observers noted a tendency for mutual-aid networks to exhibit signs of class differentiation that had perhaps been obscured by hopes that “we’re all in this together” at the start of the pandemic (Spring 2020; Tolentino 2020).

Truly mutual aid does not tinker around the edges of food systems but is predicated on a commitment to contesting them by visibilising the proximity of precarity for many and linking care-based solidarity with political demands (Guthrie 2020b; Spring and Biddulph 2020). How to link these variegated, locally-specific efforts into broader movements for systems change remains a task that will outlast the pandemic (Patel and Goodman 2020). Amidst the vast struggles faced by food sectors such as restaurants, we have also seen a resurgent ethic of sharing: of a sense that food should not be allowed to be treated as a mere commodity but as a right: not just to adequate calories but to food as a source of pleasure, hospitality and commonality (Connors et al. 2020; Healy 2021; Kuh 2020). The idea of food as a ‘commons’ has also seen a revived focus by scholars and activists (e.g. Healy et al. 2020; Jackson et al. 2021). Renewed interest in urban agriculture and land access for communities of colour has renewed the promise of radical food justice, though requires overcoming significant structural barriers (O’Hara and Toussaint 2020).

Overcoming the syndemic effects of COVID-19 and, vitally, preventing further outbreaks of ‘capitalist viruses’ (Smith 2020), cannot result from reforms to single issues or institutions. Dickinson and Lohnes (2019) consider Green New Deal proposals as ways to challenge the uneven capitalist development driving poverty and hunger, and as ways for food banks to act not in the interests of corporate donors but as nodes for well-paid job creation. Such holistic political visions have also gained traction in the UK as part of a counter-voice to populist and right-wing politics in Europe and America, evident in the 2019 Labour Party Manifesto’s articulation of an “end to ‘food bank Britain’” through a ‘Green Industrial Revolution’ (Labour Party 2019). In spite of their electoral defeats, the rise of Jeremy Corbyn and Bernie Sanders to prominence re-surfaced discussions of universal incomes and services, and of links between public housing, decent work, collective wellbeing, and democratic process. Meanwhile, an uptick in labour organising suggests efforts to “rewrite the neoliberal playbook” of ever-weaker pay and conditions alongside corporate enrichment, including by workers at some of foodbanking’s largest donors from Kellogg’s to Kroger (Kasmir 2021, p.462).

COVID-19’s encouragement of online collaboration has allowed movements, including our own, to mobilise critiques of foodbanking, grounded in experiences from different parts of the globe but recognising foodbanking’s rootedness in international food system logics (Cohen 2020). Much of this analysis, as we have shown, had been developing over four decades. However, the lasting effects of the 2008 global recession, together with the acceleration and expansion of the Global Foodbanking Network, have evolved to disperse the charitable food model around the globe. As we have outlined, the COVID-19 pandemic has led to a massive expansion of food charity, accelerating its institutionalisation and rendering collaborative contestation evermore urgent (De Schutter 2019).

## Concluding Discussion

Do antihunger responses during the COVID-19 pandemic suggest containment or contestation of inequality and poverty? We have detailed ways in which forty years of neoliberalism and austerity have positioned corporate charity as an acceptable container of household food insecurity, obscuring rights-based solutions premised on income redistribution and food justice/sovereignty. The enormous cash flows to foodbanking by corporations and governments highlighted the dominance of containment approaches to income crises, suggesting a reversal in ‘exit strategies’ by charities. However, we have also seen mobilisations around food and poverty that draw on years of contestatory movements against inequitable food systems (Lakhani 2021). Can these move beyond rhetoric towards concrete changes, in a ‘cautiously optimistic’ period when neoliberal subjectivities are being shaken by reassertions of state responsibility and the unsustainability of existing economic arrangements (Saad-Filho 2020)?

The challenges facing food providers during COVID-19 remind us that achieving greater food justice is a long process where progress is not always linear. It requires transforming not only food charities’ practices, but the contexts through which they have become (considered) necessary (Fisher 2020). Our discussion of pre-existing structural drivers of foodbanking’s internationalisation and normalisation provides such historical context as grounds for building awareness and power among food providers to abolish food and economic systems that deny the Right to Food. Abolishing harmful food systems requires investment in regenerative ones that place health over profit, including food worker fairness, market diversification, and collective revaluation of food quality and sustainability over quantity and profit (Mourad 2021). Some US foodbanks have begun to retool their procurement towards healthier and regionalised sourcing in ways that both challenge and reinforce the prominence of charitable relief (Vitiello et al. 2015); could they extend this work to consider the labour conditions inhering in the food they redistribute, for example? While putting pressure on suppliers, can food charities also foster more equitable relations between staff, volunteers and recipients, beyond simply adopting more dignified terminology- from ‘clients’ to ‘participants/members’, for example (Swords 2019)? Such internal transformation could include diversifying boards, adopting fair wage policies and dismantling systemic racism within organisations and in broader advocacy (Fisher 2020).

Our Alliance has debated whether the future of foodbanking could support transitions towards social and environmental justice. A key question remains as to whether ‘exit strategies’ from charitable expansion might entail foodbanks’ ‘retooling’ rather than their elimination. While foodbanking has been expanding globally as a way to manage contradictions of excess and hunger in industrial food systems, pressure from social movements have led some providers to reform their practice towards healthier and more dignified approaches, while others are questioning their role altogether. The complex politics at play among foodbanking spaces and actors (Dixon 2015; Henderson 2004; Williams et al. 2016) can be seen in debates over the appropriateness of the COVID charity response and over differences between charity and mutual aid. Many food providers are becoming increasingly aware of food system injustices, especially histories of racialised dispossession and enslavement (Patel and Goodman 2020). We see the influence of progressive and radical food movements on resurgent movements for reparations/land-back and BIPOC-led food sovereignty activism since the pandemic and antiracist uprisings of 2020, but it remains to see how far these can scale out to challenge the industrial food systems encouraged by dominant agrifood policy structures and globalising foodbanking industries. We hope that our paper invites further internationalist analysis and response. Solutions to food insecurity require consideration of ecology, health, and internationalism, given the globalised nature of food system interdependencies and corporate capture of global food governance (Clapp 2014; Clapp and Moseley 2020; Fakhri et al. 2021).The nature and impacts of the COVID-19 virus itself convince us of this.

## Footnotes

1 https://www.compas.ox.ac.uk/2020/not-for-the-people-pandemics-and-the-selective-foresight-of-western-neoliberal-capitalism/

2 While the term ‘foodbank’ refers to a place of direct food provision in countries like the UK, we are referring to private, networked logistical systems that redistribute surplus and donated food to local food distribution agencies, largely from private food industries. See Fisher (2017).

3 Note differences between use of term ‘food bank’ in the UK- describing where people experiencing food poverty can receive parcels of store-donated food- and the US- where it corresponds more closely to the work of FareShare UK- the logistics and warehousing of food rendered surplus throughout supply chains which is then diverted to subsidiary charities for subsequent distribution to people.

4 Researchers have examined the growth of foodbanking in places including Germany (Lorenz 2012), Korea (Kim 2015), Australia (Pollard et al. 2018) and Italy (Arcuri 2019).

5 Also described as the ‘food waste/recovery pyramid’, which prioritises responses to food wastage in terms of prevention, redistribution to humans and farmed animals, composting, waste-to-energy and landfilling; see (Marie Mourad 2016)for a critique

6 https://fareshare.org.uk/giving-food/

7 E.g. Independent Food Aid Network (n.d.) Food Bank Volunteer Hours Research https://www.foodaidnetwork.org.uk/food-bank-volunteer-hours

8 The very recruitment of staff devoted to advocacy- although what this entails varies greatly between organisations- is worthy of note, and not yet a role that FareShare, the UK’s largest US-style foodbanking organisation, includes as part of its staffing.

9 https://thehungergap.org/narrative-change-task-force/

10 For example of funds committed to local authorities as result of COVID, https://www.gov.uk/government/news/63-million-for-local-authorities-to-assist-those-struggling-to-afford-food-and-other-essentials See also (Barker and Russell 2020).

11 Also interview Christina Wong, NW Harvest, June 5 2020 (by Fisher).

12 These include tax credits, donations, cash grants, and other programmes

13 Feeding America, FRAC, Share Our Strength, Bread for the World

14 For example, the Black Church Food Security Network held a discussion on 31/05/2020, entitled Food Banks & The Faith Community: Swimming Upstream to Sustainable Solutions, questioning the rationale of food distribution alone and exploring links between systemic racism, food insecurity and corporate charity. https://www.facebook.com/watch/?v=384564782501570

15 https://fightfor15.org/

16 https://foodjustice.co.uk/open-letter
